# Exploring the Spectrum of Lymphadenopathy: Insights From a Three-Year Fine Needle Aspiration Cytology Study in a Tertiary Care Center

**DOI:** 10.7759/cureus.59049

**Published:** 2024-04-26

**Authors:** Neha Jaiswal, Sweta Bahadure, Ankit Badge, Pratibha Dawande, Vaishnavi H Mishra

**Affiliations:** 1 Pathology, Datta Meghe Medical College, Datta Meghe Institute of Higher Education and Research (DU), Nagpur, IND; 2 Microbiology, Datta Meghe Medical College, Datta Meghe Institute of Higher Education and Research (DU), Nagpur, IND; 3 Microbiology, Jawaharlal Nehru Medical College, Datta Meghe Institute of Higher Education and Research (DU), Wardha, IND

**Keywords:** metastatic lesions, cytology, reactive lymphadenitis, lymphadenopathy, fine needle aspiration cytology

## Abstract

Introduction

Lymphadenopathy, characterized by the enlargement of lymph nodes, is a common concern encountered by physicians in outpatient settings. It is deemed significant and warrants evaluation due to the diverse range of potential causes, ranging from treatable infections to incurable metastatic malignancies. Fine needle aspiration cytology (FNAC) emerges as a crucial tool in addressing these concerns, acknowledged for its rapid diagnostic capabilities, simplicity, accuracy, and minimal invasiveness.

Objectives

This retrospective study aims to characterize the spectrum of lymphadenopathy cases observed in a tertiary care center over a specified period, shedding light on the prevalence, etiology, and clinical outcomes associated with this condition.

Methods

Electronic medical records of patients presenting with lymphadenopathy to the tertiary care center between May 2021 and June 2023 were reviewed. Data on patient demographics, clinical presentation, imaging findings, and cytopathological and histopathological diagnoses were analyzed.

Results

A total of 300 cases of lymphadenopathy were identified during the study period. The study population exhibited a diverse range of age groups, with a slight predilection for the age range of 11-20 years. The most common sites of lymphadenopathy were in the cervical group, and the predominant clinical presentations included tender lymph nodes and fever.

Etiologically, infectious causes, such as accounted for the majority of cases, followed by inflammatory and neoplastic etiologies. Notably, 2.6 % of cases presented with non-specific lymphadenopathy, warranting further investigation. Diagnostic modalities, including imaging studies and histopathological examinations, played a crucial role in establishing accurate diagnoses. The study also highlights the challenges in differentiating benign from malignant lymphadenopathy, emphasizing the need for a comprehensive diagnostic approach.

Conclusion

This study provides a comprehensive overview of the lymphadenopathy spectrum in a tertiary care center, emphasizing the importance of a multidisciplinary approach for accurate diagnosis and management. The findings contribute to our understanding of the epidemiology and etiological patterns of lymphadenopathy, guiding clinicians in optimizing patient care and outcomes in a tertiary healthcare setting.

## Introduction

Lymph nodes are bean-shaped structures located along the lymph canal of the neck, axilla, thorax, abdomen, and inguinal area. Lymph nodes are collections of lymphoid cells and along with lymphatics form an important part of the immune system. Lymphadenopathy is the swelling of lymph nodes and is one of the frequent complaints encountered by physicians in the outpatient section. Any lymphadenopathy is considered significant and should be evaluated as the causes can vary from a simple treatable infection to an incurable metastatic malignancy [1,2]. The spectrum of pathological lesions of lymph nodes predominantly comprises reactive lymphoid hyperplasia, granulomatous lymphadenitis, metastatic lymph nodes, and lymphomas. While evaluating the lymph node lesion one should be aware of the common lesions that affect the lymph node in a particular geographical region [3,4]. In India, tuberculosis is quite common so any granulomatous lymphadenitis should be further investigated by acid-fast bacteria (AFB) staining [5,6]. In addition to providing a diagnosis, fine needle aspiration cytology (FNAC) is valuable for guiding early and appropriate investigations. Lymph node aspirates are typically highly cellular, and their interpretation can range from a definitive diagnosis to a strong recommendation for histopathological examination [7]. This study is undertaken to analyze and study the pathological lesions of lymph nodes in all age groups by the fine needle aspiration technique.

## Materials and methods

**Figure 1:**
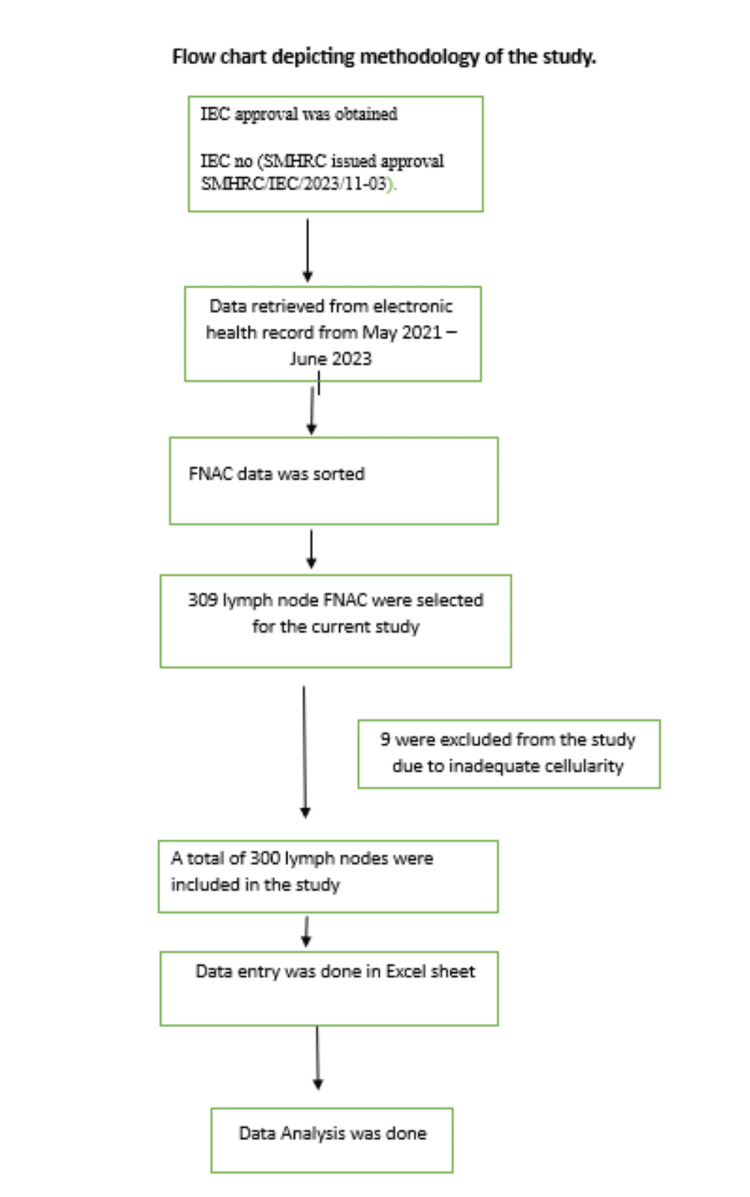
Methodology flow chart IEC: Institutional Ethics Committee; SMHRC: Shalinitai Meghe Hospital and Research Centre; FNAC: Fine Needle Aspiration Cytology

This study employed a retrospective design to analyze peripheral lymphadenopathy cases over three years from May 2021 to June 2023. The research was conducted at the Department of Pathology within the Division of Cytopathology at the Datta Meghe Institute of Higher Education and Research, located in Wanadongri, Nagpur. A total of 309 lymph node aspirates were included in the study. Patients of both genders with peripheral lymphadenopathy and adequate cellularity on microscopy were considered eligible. Cases of central lymphadenopathy and those with low cellular yield for a definitive diagnosis were excluded. The study protocol was approved by the institutional ethics committee (Figure 1). Patient confidentiality and privacy were strictly maintained throughout the study process (SMHRC issued approval SMHRC/IEC/2023/11-03).

Detailed clinical history and the results of relevant investigations were recorded for each case. A physical examination was performed following the history. History taking was followed by a physical examination. The FNAC procedure began with patient preparation, explaining the purpose and risks. Patients were positioned comfortably. Necessary sterile equipment was gathered. The lesion site was cleaned. The needle was inserted into the enlarged lymph nodes, followed by to and fro motion simultaneously applying negative pressure with the syringe to aspirate cells. The needle was withdrawn within the lesion and inserted in different directions to obtain an adequate sample. The material obtained was ejected onto the glass slides and an even smear was made with another slide. The slides were immersed into the Coplin jars containing 95% alcohol for Papanicolaou staining, while others were air-dried for Giemsa staining. Ziehl Neelson staining was done for cases suspected of tuberculosis. All the stained smears were assessed by an experienced cytopathologist. A cytology report documented the cytomorphological findings. Out of 309 smears, nine smears with scant cellularity were excluded from the study. The diagnosis was based on cytomorphological features. All neoplastic lesions were confirmed on histopathology. Upon excision, the lymph node was fixed in 10% neutral buffered formalin for 24 hours. Following fixation, grossing was done which included noting the size, consistency, and any nodules within the lymph node. The lymph node was dissected longitudinally to examine the cut surfaces for any abnormalities like nodularity, necrosis, and hemorrhage.

The sections taken were representative of both normal and abnormal areas. The tissue section underwent all processes of tissue processing including dehydration using graded alcohols, clearing, and then embedding in paraffin wax with proper orientation. The cell blocks with the embedded tissue were sectioned into thin sections of 5 mm using a rotary microtome. The obtained sections were then mounted and stained with hematoxylin and eosin (H&E) stain. The stained tissue sections were examined microscopically using various magnifications to assess the cellular composition, tissue architecture, and any pathology. Normal structures of lymph nodes, such as lymphoid follicles, germinal centers, and sinuses, were identified. The stained tissue sections were evaluated for the presence of inflammatory infiltrates, necrosis, fibrosis, or neoplastic changes. The histopathological findings were interpreted in the context of the patient's clinical history, laboratory results, and imaging studies.

Exclusion criteria

Cases of central lymphadenopathy were excluded from the analysis. Additionally, cases with low cellular yield for a definitive diagnosis were not included in the study to maintain the quality and reliability of the findings.

Inclusion criteria

Patients of both genders presenting with peripheral lymphadenopathy were included in the study, provided that cases exhibited adequate cellularity upon microscopic examination of the aspirated material. The study period spanned from May 2021 to June 2023, ensuring the inclusion of cases within this timeframe.

## Results

This study investigated a collective of 309 cases. Nine of these cases were deemed inconclusive due to insufficient cellularity and therefore were excluded from the analysis. Among men, the most prevalent pathologies are reactive 75 cases and granulomatous 74 cases, with four cases of metastatic lesions. Tuberculosis is observed in 15 cases, suppurative in 12 cases, lymphoma in two cases, and one case falls under the others category. For females, reactive pathology is predominant in 60 cases, followed by granulomatous in 46 cases and two cases of metastatic lesions. Tuberculosis is observed in six cases, suppurative in 3, while there are no cases recorded in lymphoma and other categories for women. Overall, reactive pathology constitutes 45% of cases, granulomatous pathology accounts for 40%, and the remaining categories contribute to varying proportions, offering a comprehensive overview of the pathology in both males and females. The analysis revealed that 61% of the cases of lymphadenopathy were males and 39% were females. Table 1 shows a gender-wise distribution of lymph node pathology. The Chi-Square Test is used to find the association between gender and types of lymph node lesions. According to Table 1, as the p-value is 0.3265, there is no significant association observed between gender and the distribution of types of lymph node lesions (Table 1).

**Table 1:** Gender-wise distribution of lymph node pathology n: Number; M: Male; F: Female

Gender	Reactive (n)	Granulomatous (n)	Metastatic (n)	Tuberculosis (n)	Suppurative (n)	Lymphoma (n)	Others (n)	Total (n) %	Chi Sq. Value	p-value
M	75	74	4	15	12	2	1	183 (61 %)	6.939	0.3265
F	60	46	2	6	3	0	0	117 (39 %)		
Total (n)%	135 (45%)	120 (40%)	6 (2%)	21 (7%)	15 (5%)	2 (0.66%)	1 (0.33%)	300 (100%)		

**Figure 2:**
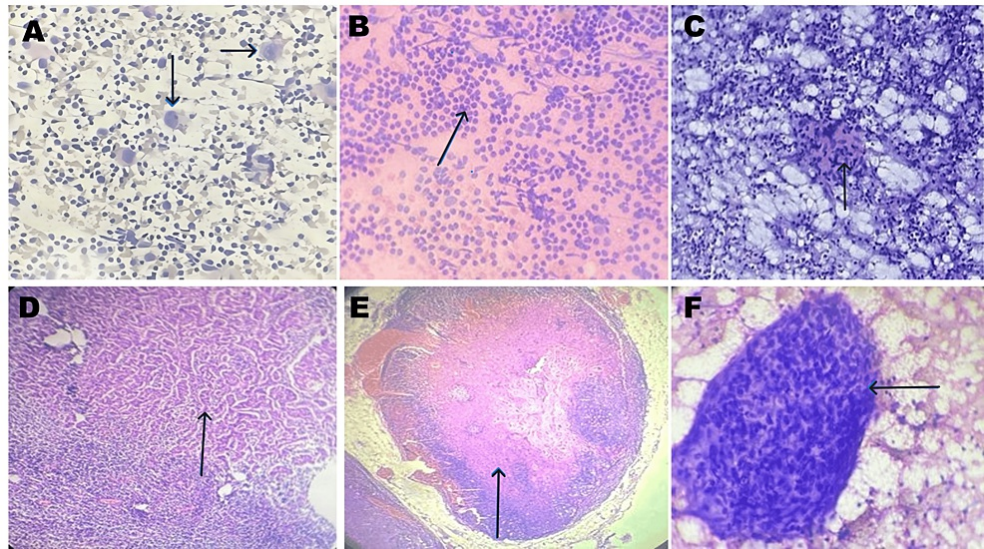
Different entities diagnosed in the lymph node A - Hodgkin’s Lymphoma in which the black arrow depicts Reed Sternberg’s Cell B - Reactive Lymphoid Hyperplasia in which the black arrow depicts polymorphous lymphocytes C - Tuberculous lymph node in which the black arrow depicts granuloma D - The black arrow depicts metastatic deposits of thyroid carcinoma in the lymph node E - The black arrow depicts metastatic deposits of squamous cell in the lymph node F - Black arrow depicts granuloma in the lymph node

Figure 2 shows different entities diagnosed in the lymph node during the study.

**Figure 3:**
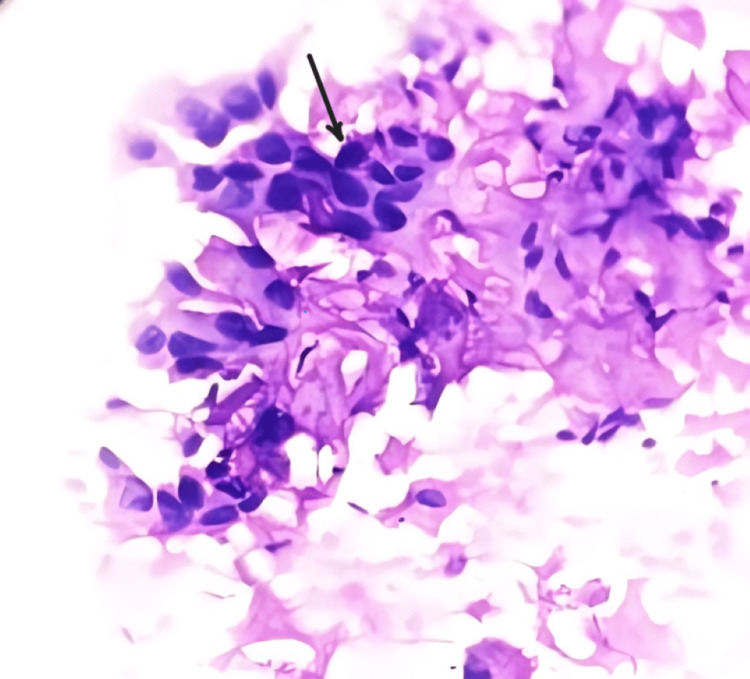
Metastasis of squamous cell carcinoma in the lymph node Black arrow - Metastatic deposits of squamous cell carcinoma

Figure 3 shows Papanicolaou's (PAP) stain metastasis of squamous cell carcinoma in the lymph node.

The cervical lymph node exhibited the highest prevalence of involvement, 258 (86%), with the following involvement observed in decreasing order: inguinal 18 (6%) and submandibular 12 (4%). A lower percentage of cases exhibit post-auricular involvement at 1%, with axillary and supraclavicular lymph nodes each contributing to 2% and 1%, respectively. The data reflect the distribution of lymph node involvement, providing valuable information on the patterns observed within the studied population (Table 2).

**Table 2:** Pathological distribution of lymph node groups n: Number of cases

Involved lymph node group	n	Percentage
Cervical	258	86%
Inguinal	18	6%
Submandibular	12	4%
Post auricular	3	1%
Axillary	6	2%
Supraclavicular	3	1%

This study included individuals aged between 7 months and 75 years. Lymphadenopathy predominantly occurred in people aged 11 to 20 years. The most common conditions observed were reactive lymphadenitis, granulomatous lymphadenitis, tuberculosis, and suppurative lymphadenitis. All cases of granulomatous lymphadenitis were negative for AFB. Twenty-one cases of tuberculosis were confirmed by positive AFB staining. Most of the cases of suppurative lymphadenitis were under 10 years. One case of necrotic lymphadenitis revealed only necrotic material. Out of 8 cases of neoplastic etiology, 6 were metastatic and 2 were lymphoma. Lymphadenopathy of neoplastic etiology was observed most frequently in individuals in their sixties and 70s. The diagnosis of metastatic lymph nodes was based on the cytomorphological characteristics of the metastatic deposits. The majority of metastatic lymph nodes were squamous cell carcinoma. Two diagnosed lymphomas were of Hodgkin’s type. The Chi-Square Test is used to find associations between age groups and types of lymph node lesions. According to Table 3, as the p-value is less than <0.0001, age is strongly correlated with the distribution of types of lymph node lesions (Table 3).

**Table 3:** Age-wise distribution of lymph node lesions n: Number of cases

Age	Granulomatous lymphadenitis (n)	Reactive lymphadenitis (n)	Tuberculosis (n)	Suppurative lymphadenitis (n)	Metastatic (n)	Lymphoma (n)	Others (n)	Total	Chi Sq. Value	p-value
0 – 10	3	30	0	9	0	0	0	42	175.73	< 0.0001
11 – 20	62	27	4	3	0	0	0	96		
21 – 30	18	24	2	1	0	0	0	45		
31 – 40	16	12	5	0	0	0	0	33		
41 – 50	11	18	6	0	0	0	1	36		
51 – 60	7	15	0	2	2	1	0	27		
61 – 70	3	5	4	0	3	0	0	15		
>70	0	4	0	0	1	1	0	06		
Total	120	135	21	15	06	02	01	300		

Out of six cases of metastatic lymph node, four cases were of squamous cell carcinoma, one was of infiltrating ductal carcinoma, and one was of thyroid carcinoma. All squamous cell carcinomas were of oral origin. Cytological and histopathological co-relation on metastatic malignancy in lymph nodes (Table 4).

**Table 4:** Cytological and histopathological co-relation on metastatic malignancy in lymph nodes n: Number of metastatic lesions

Metastatic lesion	n	Cytology	Histopathology
Squamous cell carcinoma	04	Squamous cell carcinoma	Squamous cell carcinoma
Infiltrating Ductal carcinoma	01	Infiltrating Ductal carcinoma	Infiltrating Ductal carcinoma
Thyroid Carcinoma	01	Thyroid Carcinoma	Thyroid Carcinoma

## Discussion

Ha et al. initially introduced the technique of FNAC. FNAC has become widely used as a diagnostic tool for rapid evaluation of mainly superficial lesions [8]. Lymphadenopathy refers to lymph nodes exhibiting abnormalities in size, consistency, or number. It is one of the most frequent and clinically significant complaints encountered by a physician in an outpatient department daily [9]. Lymphadenopathy manifests itself as a clinical indication of regional or systemic disease, providing valuable clues about the underlying health condition. In certain cases, cervical lymphadenopathy may represent the only clinical observation [10]. The fine needle aspiration technique is a simple, rapid, and cost-effective outpatient department procedure that can be employed for assessing the cause of lymphadenopathy [11-13]. The two most common percutaneous sampling methods are FNA and core needle biopsy. Core biopsy is typically preferred when neoplasia is suspected in a lymph node as it provides a larger tissue sample and also provides architectural information. FNA is less invasive and allows immediate confirmation of adequacy by the attending cytologist [14].

The diagnostic sensitivity, specificity, positive predictability, and negative predictability of the lymph node needle aspiration technique (FNAC) were 87.9%, 100%, 100%, and 89.7%, respectively. The overall diagnostic accuracy was 94.1%. The reported accuracy of lymph node FNAC in existing literature ranges from 82% to 94.4% [15]. In the diagnosis of suspected lung cancer, excision biopsy of supraclavicular lymph nodes showed 100% sensitivity, 96.7 % specificity, 83.3 % positive predictive value, 100 percent negative predictive value, and 97.8 % diagnostic accuracy [16]. As FNAC is less invasive and has fewer complications, it can be a safe alternative to excision biopsy and should be recommended as a first-line investigation while excision biopsy as a second-line investigation only if the results of FNAC are not satisfactory [17].

FNAC can be used as a substitute for excision biopsy in many instances [18]. However, ambiguities persist in achieving precise diagnoses, particularly in cases of primary lymphoproliferative disorders. Differentiating between low-grade non-Hodgkin lymphoma and reactive hyperplasia can present a diagnostic challenge, even for experienced professionals [19]. Excisional lymph node biopsy proves highly effective, particularly in delineating lymphoma subgroups for initiating oncologic treatment [20].

We studied 309 cases of lymphadenopathy retrospectively and prospectively for three years. Nine cases were excluded from the study due to inconclusive results due to low cellularity in the smears. Among the remaining 300 cases, 98% were identified as non-neoplastic, while 2% were classified as neoplastic. The non-neoplastic lesions included granulomatous lymphadenitis, reactive lymphadenitis, tuberculous lymphadenitis, and suppurative lymphadenitis. Neoplastic conditions included metastatic squamous cell carcinoma, metastatic breast infiltrating ductal carcinoma, metastatic thyroid carcinoma, and Hodgkin lymphoma.

In this study, the cervical lymph node was the most commonly affected (84%), with the inguinal and submandibular nodes following in frequency. A study by Gayathri et al., [21] supported these results, highlighting the cervical group as the most commonly affected, consistent with our findings. According to the present investigation, the age group most frequently affected by lymphadenopathy was between 11 and 20 years old. Granulomatous lymphadenitis occurred predominantly in individuals aged 11 to 20 years, while reactive and suppurative lymphadenitis was observed in children under the age of 10. Metastatic lymphadenopathy was observed in the older age group between 50 and 70 years.

The study by Bansal et al. [22] showed that of 100 lymph node examinations 30 were found to be granulomatous, 27 were found to be reactive, 19 were suppurative, and 12 were neoplastic. Out of the 12 neoplastic lymph node aspirates, 8 were metastatic and 4 were lymphoma. In the current study, the most frequently diagnosed condition is reactive lymphadenitis, followed by granulomatous lymphadenitis, consistent with the findings of a study conducted by Gayathri et al., [21] on 1774 lymph node aspirates. Most of the patients with reactive lymphadenitis were in the age group of 11 to 20 years. The study by Ashwini et al., [23] and Tiwari et al., [24] revealed that most patients with lymphadenopathy were cases of tuberculosis which is not consistent with the present study. The study conducted by Ashwini et al., [23] revealed that out of 310 lymph nodes 151 were tuberculosis, 109 were granulomatous and 15 were neoplastic (12 metastatic, 03 - Lymphoma). The study conducted by Attaullah et al., [25] found that of 235 cases of lymphadenopathy, 110 cases were of granulomatous lymphadenitis, which is in concordance with the present study.

In our investigation, the cases of granulomatous conditions were characterized by the presence of granulomas, consisting of epithelioid cells, histiocytes, and lymphocytes. Notably, these cases tested negative for acid-fast Bacilli on ZN stain. In contrast, cases that tested positive for AFB were identified as tuberculosis. Our study documented 120 cases (40%) as granulomatous due to similar cytological features. The purative cases, on the other hand, exhibited only neutrophil sheets without the presence of granulomas. Specifically, cases of squamous cell carcinoma demonstrated pleomorphic tumor cells showing a hyperchromatic nucleus, eosinophilic cytoplasm, and concentric layers of keratinized cells known as keratin pearls, which are often observed in well-differentiated squamous cell carcinoma.

On comparing the results of our study with previous research, several significant observations were made. Primarily there is a considerable difference in sample size. Our study examined 300 lymph nodes, While Bansal et al. [22] did a study on 100 lymph nodes, Gayathri et al. [21] studied a larger sample of 1774 lymph nodes. Distinctive variation can be observed in the results of the other studies when compared with the present study. Our study revealed a high prevalence of granulomatous lymphadenitis, similar to Bansal et al. [22], which reported 30 cases of granulomatous lymphadenitis out of 100 cases of lymphadenopathy. Reactive lymphadenopathy appears to be a consistent finding across studies, with similar proportions reported in our study (135 cases) and Gayathri et al., [21] (465 cases). However, Tiwari et al. [24] notably observed lesser cases of reactive lymphadenopathy.

The prevalence of specific etiologies of lymphadenopathy also varies among studies. Tuberculosis features prominently in our study (21 cases) as well as in the studies by Ashwini et al. [23] and Tiwari et al. [24]. Conversely, Lee et al. [16] reported a higher incidence of metastatic lymphadenopathy (376 cases), which was not observed in our study. These discrepancies may stem from variations in geographical location or patient demographics. Additionally, the incidence of inconclusive diagnoses varies across studies, suggesting challenges in definitive pathological interpretation. While our study reported six inconclusive cases, Bansal et al. [22] and Tiwari et al. [24] also encountered cases where a conclusive diagnosis could not be reached.

In summary, comparing findings across multiple studies highlights the importance of considering various factors such as sample size, population demographics, and diagnostic methodologies when interpreting pathological entities. Further research with larger sample sizes and standardized diagnostic criteria may offer additional insights into the epidemiology and etiology of lymphadenopathy.

Limitations

To improve the representation of the community, it is necessary to have a larger sample size and an extended study duration. A comparison of the present study with other studies is shown in Table 5.

**Table 5:** Comparison of the present study with other studies

Present study	Total number of lymph nodes studied	Granulomatous	Reactive	Tuberculosis	Suppurative	Metastatic	Lymphoma	Others	Inconclusive
Present study	300	120	135	21	15	06	02	01	06
Lee et al. [16]	1774	262	465	260	70	376	13	-	328
Bansal et al. [22]	100	30	27	04	19	08	04	02	-
Ashwini et al. [23]	310	109	25	151	05	12	03	-	05
Tiwari et al. [24]	163	-	45	67		16	08	27	-
Attaullah et al. [25]	235	110	108	-	-	05	10	02	-

## Conclusions

The prevalence of lymphadenopathy within the studied population mirrors and, in some cases, challenges existing literature. A diverse range of age groups participated, with a nuanced predilection for the 11-20-year age group. The FNAC lymph node proves to be an effective method of diagnosing both inflammatory and neoplastic lesions. The cost-effectiveness and simplicity contribute to increased patient compliance. The insights can guide clinicians in tailoring diagnostic strategies, urging earlier interventions, and optimizing treatment plans. By understanding the diverse spectrum of lymphadenopathy, healthcare providers can improve patient outcomes and streamline resource allocation in tertiary care settings.
